# Exploring the feasibility of conducting a randomised controlled trial of group-based pregnancy care and education: a pilot randomised controlled trial in Melbourne, Australia

**DOI:** 10.1186/s40814-024-01501-8

**Published:** 2024-05-20

**Authors:** Della A. Forster, Robyn Matthews, Rebecca Hyde, Deborah Fox, Kaye Dyson, Trish Ryan

**Affiliations:** 1 Judith Lumley Centre, La Trobe University, Bundoora, VIC 3086 Australia; 2 School of Nursing & Midwifery, La Trobe University, Bundoora, VIC 3086 Australia; 3 The Royal Women’s Hospital, Locked Bag 300, Cnr Grattan St and Flemington Rd, Parkville, VIC 3052 Australia; 4 Centre for Midwifery, Child and Family Health, University of Technology Sydney, Ultimo, NSW 2007 Australia

**Keywords:** Randomised controlled trial, Antenatal care, Pregnancy, Group care, Antenatal education

## Abstract

**Background:**

In group-based pregnancy models, antenatal care and childbirth/parenting education are provided in groups of eight to 10 women, usually with two midwives, and six to eight sessions. Current evidence is inconclusive regarding potential benefit or harm. We aimed to explore the feasibility of implementing an adequately powered randomised controlled trial (RCT).

**Methods:**

A two-arm pilot RCT was conducted in a tertiary maternity hospital in Melbourne, Australia. Women were randomly allocated to either the intervention to receive group-based antenatal care and education (group care) or to usual care, which included hospital-based midwife, caseload midwifery, team midwifery, or GP shared care. Participants were English-speaking, primiparous, low risk, and < 24 weeks gestation at booking. Data collection: feasibility measures throughout pilot, baseline questionnaire at recruitment, clinical outcome data from the medical record, and a telephone-administered questionnaire 6 weeks postpartum. A focus group explored midwives’ views.

**Results:**

Seventy-four women were recruited from May to June 2017 (group care = 40, usual care = 34). Study uptake was 35%. Women allocated to group care rated their overall pregnancy care more highly (88% good/very good vs 77% in usual care). There was no evidence of harm related to group care. Overarching themes from the midwives were that group care helped ‘build connections’ and ‘empower women’. All midwives would work in the model again and believed it should be expanded.

**Conclusion:**

Group care was acceptable to both women and midwives with no evidence of harm. The pilot demonstrated the feasibility of undertaking a large adequately powered RCT, important given the inconclusive evidence on clinical outcomes regarding the model, and its current relatively widespread implementation.

**Trial registration:**

Australian New Zealand Clinical Trials Registry (ANZCTR): ACTRN12623000858695.

**Supplementary Information:**

The online version contains supplementary material available at 10.1186/s40814-024-01501-8.

## Key messages regarding feasibility

*• What uncertainties existed regarding the feasibility?* Both women and midwives are positive about group-based pregnancy care, but more trial evidence is needed to test safety and efficacy. We planned to undertake a randomised controlled trial (RCT) in Melbourne, Australia, but did not know if would be possible to develop and implement group-based pregnancy care in such a way as to allow scale-up for a larger adequately powered RCT. We needed to explore if midwives were willing to implement the model, and if so, whether women would be willing to have this model of care for their pregnancy journey, and if so, if they would be willing to be randomised. We also needed to know what percent of women at our potential trial site would be eligible, and of these, what percent would consent and participate. Other aspects we needed to ascertain were if the site infrastructure and facilities could accommodate group-based care, and if we could obtain clinical outcome data in a timely manner.

*• What are the key feasibility findings?* This pilot RCT demonstrated the feasibility of undertaking a large adequately powered RCT in the Australian context. We developed and piloted our group-based pregnancy care schedule, and recruited eight midwives to provide the group-based care. We were able to identify appropriate rooms within the hospital to conduct the sessions and book them for the entire period they were needed. We found women who were willing to participate in the study and to be randomised, and we were able to assess study uptake and the proportion of eligible women. Clinical outcomes were obtained, there were no signs of obvious harm, and we were able to use the data in inform sample size calculations for a larger RCT. Women's and clinicians' views were obtained, and these were all very positive—both groups really supported the concept of the group-based care. The other key outcome related to feasibility was bringing together an investigator team (including a consumer who had been a participant in the pilot and received group care) to be part of a grant application for the larger study.

*• What are the implications of the feasibility findings for the design of the main study?* We conclude that the planned larger adequately powered RCT is feasible, and that there are only minor adjustments that need to be made based on our pilot findings. Almost all women brought a support person with them to the groups, and very few preferred women-only sessions, which we would take into account in designing the larger study. Some women would have liked the option of their sessions being held on the weekend, and some would be happy to attend a community-based setting—and these are aspects we can also plan for in the larger study. In terms of the midwives, the main issue was finding time for the administrative tasks, so this is something we need to plan for in the larger RCT, and measure in the process evaluation.

## Background

Pregnancy and birth provide a critical window for intervening to improve short- and long-term health and well-being for women and their children, yet there is a lack of evidence to guide preventative interventions. It is therefore critical to identify models that have a positive impact on clinical outcomes, are acceptable to women and providers, and are scalable and sustainable. We were interested in exploring midwife-led group-based pregnancy care and education (Group Care) to address some key outcomes associated with significant maternal and neonatal morbidity, such as the proportion of births by caesarean section (CS), and the proportion of infants born premature or low birthweight. All three are associated with significant maternal and neonatal morbidity [1, 2], yet there has been very little progress on improving these in recent decades.

Midwife-led group antenatal care and education (Group Care) integrates antenatal care, childbirth preparation, and early parenting education into group sessions that occur at regular intervals throughout pregnancy, facilitated by two midwives who remain with the group (as compared to standard individual-based care) [3]. Group Care incorporates two approaches shown to improve clinical outcomes and decrease unnecessary interventions—continuity of midwife care [4], and focused education [5]—combined with peer social support to provide normative guidance and increased motivation for self-care and help-seeking [6], and increased *information* sharing about childbirth, breastfeeding, and early parenting [7]. The aim is to help women to choose health-promoting behaviours, and be active participants in their care [3].

The 2015 Cochrane systematic review of Group Care shows that this model of care is acceptable to women, without obvious adverse outcomes [3]. It is also popular with midwives [8, 9]. A systematic review from 2016 included a new cluster RCT from the USA (and 10 observational studies, nine from the USA and one from Canada), and found that while the observational studies showed a lower rate of low birthweight, the RCT did not [10]. A 2017 systematic ‘overview’ found that while several groups of high-risk pregnant women may have benefits from Group Care, there was a lack of high-quality studies [11]. A systematic ‘evidence synthesis’ from 2018 stressed that implementation should be accompanied by robust evaluation [12]. Only the Cochrane review reports on CS–RR 0.83 (95% CI 0.68, 1.02), but the total number in the analysis is 842, so is likely underpowered. A new retrospective cohort study from the USA (*n* = 621) found fewer CS births for women in group care (14 vs 25%, RR 0.56, 95% CI 0.39, 0.82) [13]. Three RCTs are currently underway—in the UK [14], the Netherlands—a stepped wedge cluster design (despite no evidence of efficacy or harm) with neonatal and maternal morbidity as the primary outcome [15], and the third in the USA exploring the effect of Group Care on racial disparities in preterm birth in a very specific population [16]. The World Health Organization considers Group Care a feasible pregnancy care option, but only in the context of rigorous research—not otherwise [17].

Group Care is increasingly widespread, including in Australia [8], despite lack of evidence for both efficacy and potential harm. We planned to implement and evaluate midwife-led, group-based pregnancy care in an adequately powered RCT, but first needed to explore if this was feasible, so conducted a pilot RCT to investigate this. Our pilot study is reported in this paper.

## Methods

### Design

Two-arm randomised controlled pilot RCT.

### Primary aim

Develop and implement the Group Care model, and pilot test it against the standard schedule of individual visits, to assess if it is feasible to test the model in an adequately powered RCT.

### Specific objectives

1. Develop Group Care schedule
2. Implement pilot RCT and check all processes
3. Ascertain (a) whether women are interested in participating in the pilot, and if so, (b) if they are willing to be randomised to receive the Group Care model or usual care
4. Assess uptake
5. Check for any obvious evidence of harm (compare clinical data in trial arms, but sample will be too small to explore associations)
6. Develop primary clinical outcome/s for the larger RCT (explore what is feasible and most appropriate, e.g. type of birth, pregnancy gestation)
7. Collect data to inform sample size calculations for the proposed larger RCT
8. Explore women’s and clinicians’ views.

### Setting

The Royal Women’s Hospital in Melbourne, Australia, which has an annual birth rate of approximately 7700 births.

### Participants

#### Inclusion criteria

English-speaking primigravid women with a singleton uncomplicated pregnancy who met the hospital’s clinical guidelines as low obstetric risk at the time of the booking visit (i.e. eligible for midwife-led care) and who were ≤ 24 weeks pregnant at recruitment were eligible.

#### Exclusion criteria

Women were ineligible if (at the booking visit) they had a high level of social risk or vulnerability (needing specialised one-to-one support in pregnancy), had drug and alcohol issues that required specialist care, were experiencing significant mental health issues, or were unable to provide informed consent.

### Usual care

Women in both groups had care per the study site pregnancy clinical guidelines. All had access to support services normally available to them, and medical input as appropriate.

Women randomised to usual care could choose from all the standard low-risk care options at the study site: caseload midwifery care (one-to-one continuity midwifery care throughout pregnancy, birth and postnatally; subject to availability), team midwifery (a small team of about eight midwives who are rostered in antenatal, birth, and postnatal areas who provided care to a group of women; subject to availability), standard midwife care (no continuity but midwives provide majority of care), and shared care (the hospital shares the pregnancy care with a general practitioner, or less commonly with a private obstetrician or midwife).

Antenatal education classes were offered in traditional group-based interactive workshops with class sizes averaging 12 couples (or woman and her support person). The workshops are provided at a small cost for the woman (except if there is social disadvantage and the woman is unable to pay), and are usually booked out months in advance, and limited to women having their first baby. Not all women who are interested are able to obtain a place. Approximately 55% of first-time mothers booked to birth at the study site usually attend an antenatal education workshop.

### Intervention

Women allocated to Group Care received all their antenatal care in small groups of approximately 10 women of a similar gestation. They were offered a choice of group times and locations based on their due date, with each group including women due in the same two-to-three-week period to maximise peer support potential, and optimise timing of education and discussions. There were pre-set appointment schedules, and no waiting time for pregnancy visits. Group session dates and times were booked for a woman’s entire pregnancy. Groups met for 2 h, six times in pregnancy, at the standard pregnancy appointment gestations. Each group was run by the same two midwives every session, to provide continuity and to allow flexibility with information provision over the course of any particular group, so that timing of information/discussions could be altered to suit group needs. All sessions included routine clinical care and assessment, along with childbirth education, preparation for parenting, and peer support. Physical assessments, e.g. fundal height and fetal heart rate, took place individually, in a private screened area in the group space to maintain privacy (which also provided some one-on-one time with the midwife). Emphasis was placed on engaging women in their own health care and empowering them to be proactive [3]. Women could bring a partner or support person, with some groups available for women only. The supplementary table summarises the differences in the models. Women randomised to the intervention who experienced changes in their risk status during pregnancy that required additional obstetric care, or care from other health professionals, remained in the group, with obstetric or other care as required.

The schedule of group sessions was based on the pregnancy low-risk guideline used at the time of the study, and scheduled for 26, 30, 33, 38, and 40 weeks’ gestation. An optional weekend session was offered at 32 weeks to allow partners/support people (who may not have been able to attend during the week) to attend, and to provide further education regarding labour and breastfeeding and a hospital tour. All women, regardless of model of care, had a routine individual appointment at 36 weeks with a doctor (standard practice at the study site at the time). If the woman remained pregnant after 40 weeks, an extra individual appointment with a doctor was organised. If a woman was unable to attend a group session, an individual appointment was organised within a fortnight to ensure that the woman did not miss out on care. Where possible, the appointment was made with one of her group facilitators, to ensure continuity.

### Midwife recruitment and training

A 2-day training course included a combination of theory and ‘hands on’ learning, and introduced the basic principles of group-based antenatal care and how this differs from traditional methods of delivering antenatal care and childbirth education. The skills required for leading and facilitating a group session were explored. An expression of interest process sought midwives who provided antenatal care and/or childbirth education at the study site. Eight midwives were recruited and placed into pairs to facilitate groups throughout the pilot. Each pair had at least one midwife with previous experience in childbirth education and one with recent experience in providing pregnancy care. The research team met with the midwives to ensure all were confident in providing antenatal care through this model. To optimise continuity during the pilot, the midwives chose not to take planned leave, and if sick leave occurred, a midwife from another group helped conduct the session to ensure the group facilitation method was consistent.

### Recruitment to pilot

Research midwives invited consecutive eligible women attending their pregnancy booking appointment to participate in the study. Women who wanted to participate provided written consent, completed a baseline questionnaire, then were randomly allocated to group care or standard pregnancy care (usual care). Women allocated to group care received their schedule of visits, including dates and times. Women in usual care received subsequent appointment times via the ward clerks as per usual practice.

### Randomisation procedure

A paper-based system of randomisation was designed for the pilot. An independent researcher generated a random allocation sequence for each of the proposed groups, and individual allocations were sealed in sequentially numbered opaque envelopes and stored in a locked cabinet. To obtain a woman’s allocation, the research midwife opened the next sequentially numbered envelope and identified group allocation. The woman’s details were recorded, including the woman’s hospital record number and name, and each was assigned a unique study identification number (study ID).

### Sample size

This was a pilot whose main aim was to explore if it was possible to develop and implement the group care model at the study site, if women would take it up, and if so, if they would be willing to be randomised. We therefore did not do power calculations based on a clinical outcome. Instead, we based our sample size on recruiting enough women to assess feasibility. We estimated that running four groups of 10 women would be adequate to ascertain RCT potential uptake, as well as look at limited clinical outcome data to check for any obvious potential harm resulting from participating. Four groups was considered sufficient to provide adequate information about the proportion of women who attended the entire ‘course’ of group care sessions, whether we could provide the necessary infrastructure and staffing to run the groups at the site, and also to gain an understanding of what proportion of women would choose to have their partner/support person with them at such a group. We therefore aimed to recruit 80 women to the pilot RCT, expecting approximately half would randomly be allocated to group care.

### Data collection

Feasibility outcomes are listed in Table 1 below, summarised by both measure used and progression criteria requirement/s.

**Table 1:** Feasibility measures and progression criteria

Feasibility measure	Progression criteria
Intervention developed and implemented	Clinical leadership team approves schedule Group care model implemented (including recruitment of midwives)
Percentage of women eligible	Require 20% of women booked at potential site to be eligible
Percentage of women who consent to randomisation (study uptake)	Require minimum 20% study uptake
Retention in group care groups	More than 6/10 remain in group care group for 70% of groups
Satisfaction with group care	Overall rating of pregnancy care at least as high as standard care.^a^
Outcome data collection – birth outcomes	Require minimum 90% of birth outcome ascertainment
Outcome data collection – 6 weeks postpartum	Prefer 70% response rate to measure secondary outcomes
Midwives’ views	Need to be able recruit staff to model and ensure satisfaction with this way of working

^a^Rating of overall care in pregnancy as ‘6’ or ‘7’ on a 7-point scale where ‘7’ was ‘Very good’ and ‘1’ was ‘Very poor’

Baseline data were collected at recruitment and included demographic questions (e.g. age, smoking status, height, weight, English-speaking ability), planned model of care if not allocated to Group Care, and breastfeeding intentions.

Clinical data were abstracted from the electronic obstetric record following birth, blinded to group allocation and included standard clinical outcome data, e.g. gestation, type of birth, complications, and maternal and neonatal morbidity and mortality.

Women’s experiences and satisfaction were explored via a questionnaire conducted by telephone 6 weeks postpartum.

The questionnaire used data tools from the team’s previous studies [18] [19]. Questions explored women’s satisfaction with pregnancy care, number of appointments, waiting times, and support people’s attendance at appointments. A series of statements using five-point Likert-type scales ranging from ‘Strongly disagree’ to ‘Strongly agree’ explored women’s experiences pregnancy care, such as if women felt listened to, if their worries/anxieties taken seriously, and if the information provided was adequate. Specific questions for women allocated to Group Care explored group day and time preferences, adequacy of venues, privacy, group attendance, and other aspects, e.g. if they would recommend it to others, and how they felt about discussion of sensitive issues. All women were asked to rate overall care in pregnancy, labour, and birth and the postnatal period on a seven-point scale ranging from ‘Very poor’ to Very good’ [18], and women’s confidence and preparation for caring for their baby and parenting were explored. The Edinburgh Postnatal Depression Scale (EPDS) [20] explored women’s emotional well-being. Data were collected on breastfeeding and confidence with parenting at the time of the interview. Some questions allowed further comment.

The survey was piloted with colleagues, then with postpartum women who were not in the study. Minor amendments were made as required, then the survey re-piloted and final corrections made.

#### Clinicians’ views

A focus group was conducted with the midwives who provided the Group Care at the completion of the intervention period. The focus group was conducted by *RH* and *RM*, and explored why the midwives volunteered for the study, any concerns, any positives, and any suggested changes. The group was audio-recorded and transcribed verbatim.

### Data management and analysis

Participant responses were recorded on paper-based surveys, then the data entered onto an electronic web-based collection program, REDCap [21]. Clinical data were provided in Excel spreadsheets. All the data were imported into STATA Version 14 [22] for cleaning and analysis. Data cleaning included checks for missing data, and range and logic checks. Any discrepancies in the data were checked and the outcome agreed by members of the research team. Descriptive analyses were used for quantitative data using frequencies and percentages, and for continuous data, means and standard deviation (SD), and the outcomes shown by group allocation. Statistical comparisons between groups were not undertaken given this was not the study aim. The nature of the pilot necessitated non-blinding of participants, but all comparative data analyses were undertaken blinded to group status, and wherever possible data collection was also blinded to group.

The midwives’ focus group was analysed using simple thematic analysis (RH and RM). The transcript was first read and re-read by each separately, and simple codes abstracted from the text. Their codes were compared and agreed on, then categories developed from the codes, followed by grouping the categories into emergent themes to underpin the overarching concept/s from the text.

## Results

**Fig. 1:**
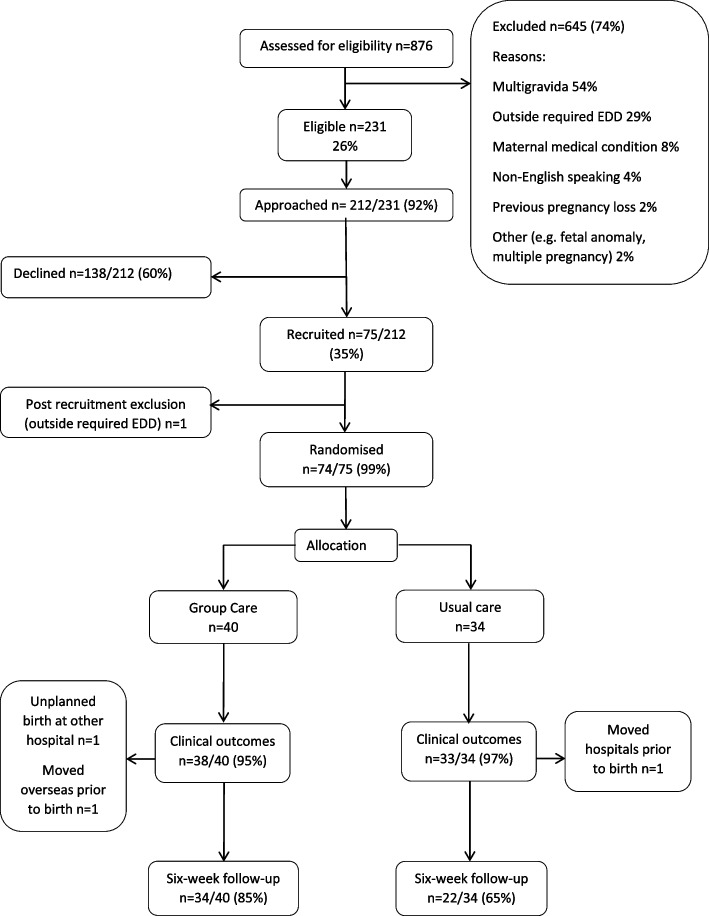
Participant flow through study

Women were recruited between May and June 2016. During this time, 26% (230/876) of women were eligible, and 35% (75/212) of those approached agreed to participate (Fig. 1). Reasons for declining included being unsure (31%, 43/138), preferring another model of care (28%, 38/138), and not wanting care in a group setting (27%, 37/138). No women declined because other people’s partners would be present or because they would be randomised.

By chance, 40 women were randomly allocated to group care by the time 75 women were recruited, so recruitment ceased then. One woman recruited was not randomised (ineligibility discovered pre-randomisation), leaving 34 women allocated to usual care. Two women’s clinical outcomes were not available because one birthed elsewhere and one moved overseas just prior to birth. On average, the follow-up interview after the birth was conducted at 8.7 weeks postpartum (range 6 to 16 weeks) and was completed by 85% of women allocated to Group Care (34/40) and 65% of women allocated to usual care (22/34).

### Feasibility outcomes

The overall aim of the pilot was to assess the feasibility of testing the Group Care intervention in an adequately powered RCT. Table 2 summarises the feasibility measures and outcomes, and demonstrates that across all measures it is feasible to continue to the larger RCT.

**Table 2:** Feasibility measures and outcomes

Feasibility measure	Progression criteria	Outcome	Comment
Intervention developed and implemented	Clinical leadership team approves schedule Group Care model implemented (including recruitment of midwives)	Model and schedule approved Midwives recruited to the model Four Group Care series conducted	Feasible organisationally
Percentage of women eligible	Require 20% of women booked at potential site to be eligible	26% were screened as eligible	Higher percentage eligible than required – shows feasibility
Percentage of women who consent to randomisation (study uptake)	Require minimum 20% study uptake	35% of eligible women approached consented to study	Meets feasibility criteria
Retention in group care groups	More than 6/10 remain in group care group for 70% of groups	Very few women missed more than one session	Retention in group care model very high, showing feasibility
Satisfaction with group care	Overall rating of pregnancy care at least as high as standard care.^a^	75% in standard care rated pregnancy care highly vs 88% in group care	High satisfaction rating with group care model – supports RCT
Outcome data collection – birth outcomes	Require minimum 90% of birth outcome ascertainment	Birth outcomes obtained for 96% of women	Meets feasibility requirement
Outcome data collection – 6 weeks postpartum	Prefer 70% response rate to measure secondary outcomes	76% overall response rate to postpartum survey	Meets feasibility requirement
Midwives’ views	Need to be able recruit staff to model and ensure satisfaction with this way of working	All midwives extremely positive about providing group care	Meets feasibility requirement

^a^Rating of overall care in pregnancy as ‘6’ or ‘7’ on a 7-point scale where ‘7’ was ‘Very good’ and ‘1’ was ‘Very poor’

Table 3 describes the women’s characteristics and demonstrates that the groups were similar.

**Table 3:** Women’s characteristics at recruitment

Characteristic	Usual care		Group Care	
	***n***	%	***n***	%
	(*n* = 34)		(*n* = 40)	
Maternal age at recruitment (years) mean (SD)	31.6	*3.2*	31.8	*4.2*
Married/living with partner	34	*100*	38	*95*
Education—degree or higher	28	*82*	31	*78*
Household weekly income pre-tax				
≤ $1999	7	*21*	14	*35*
≥ $2000 to $2999	23	*68*	20	*50*
Declined to answer	4	*12*	6	*15*
Pension or benefit	0	*0*	1	*3*
Born in Australia	14	41*	19	48*
English first language	24	*71*	25	*63*
Smoked pre-pregnancy	5	*15*	1	*3*
Maternal BMI pre-pregnancy (*n* = 32, 37)				
Overweight/obese (BMI ≥ 25)	12	*38*	13	*35*
Planning to breastfeed six months or more	23	*68*	25	*63*
Planning to attend childbirth education classes (prior to randomisation)	32	*94*	40	*100*
Model of care (usual care only, *n* = 33)			-	-
Caseload	7	*21*	-	*-*
Team midwifery	8	*24*	-	*-*
Standard midwifery care (no continuity)	16	*48*	-	*-*
Medical care	1	*3*	-	*-*
Not documented	1	*3*	-	*-*

*Other countries of birth (most across both groups): UK, Ireland, NZ, Argentina, Bangladesh, Chile, China, Colombia, East Timor, Ethiopia, France, India, Italy, Japan, Malaysia, Philippines, Samoa, Singapore, Sri Lanka, Taiwan, Turkey, Zimbabwe

### Clinical outcomes

The clinical outcomes were similar overall by group allocation, although there was a higher percentage of women in Group Care who had epidural analgesia for labour and whose babies had infant formula during the hospital stay (Table 4).

**Table 4:** Clinical outcomes

Clinical outcomes	**Usual care**		**Group Care**	
	***n***	%	***n***	%
	**(*n* = 33)**		**(*n* = 38)**	
Labour and birth				
Onset of labour – spontaneous	17	52	20	53
Epidural analgesia for labour	15	45	25	66
Caesarean birth	10	30	11	29
Baby gestation at birth (weeks)				
< 37	1	3	3	8
≥ 37	32	97	35	92
Birthweight (grams)				
< 2500	1	3	1	3
≥ 2500	32	97	37	97
Infant admitted to neonatal/special care unit (*n* = 33, 37)	3	9	5	14
Received infant formula since birth, before discharge (*n* = 29, 35)	6	21	12	34

### Women’s views and experiences of care in pregnancy

Women in usual care reported having 9.2 pregnancy appointments on average compared with 10.5 for women in group care. Almost half the women in group care (47%, 16/34) reported never having to wait for a midwife appointment, whereas half the women in usual care (50%, 11/22) reported waiting ≥ 30 min on average.

Initially, two of the four groups were planned as women-only groups. Recruitment to those groups was difficult, so they were changed to include partners. In Group Care, 67% of women (22/33) reported that their partner/support person attended five or more appointments, compared with 50% of women in usual care (11/22). The most common reason a partner/support person did not attend appointments was work commitments (both groups). Fifty-five percent of women in usual care reported that their partner/support person was encouraged to come to appointments compared with 76% of women in Group Care. Sixty-four percent of women in usual care felt that it was important that their partner/support person attended appointments compared with 85% of women in Group Care.

Table 5 shows women’s views of pregnancy care.

**Table 5:** Women’s views of pregnancy care

Statement	Agree/strongly agree^a^ Usual care *n* = 22		Agree/Strongly agree^a^ Group care *n* = 34	
	*n*	%	*n*	%
At my check-ups I was always asked if I had any questions	20	*91*	32	*94*
I was always given an active say in decisions	21	*95*	27	*79*
I always felt my worries anxieties or concerns about the pregnancy and baby were taken seriously by the midwives	19	*86*	34	*100*
I always felt my worries anxieties or concerns about the pregnancy and baby were taken seriously by the doctors	18	*82*	26	*76*
At my check-ups the midwives often seem rushed	2	*9*	4	*11*
At my check-ups the doctors often seemed rushed	4	*18*	10	*29*
I was always listened to	21	*95*	29	*85*
I got adequate information during my pregnancy about caring for my baby	14	*64*	28	*82*
The way the information was provided to me in my pregnancy was satisfactory	16	*73*	31	*91*
All my questions were answered during my pregnancy	19	*86*	31	*91*
I felt some topics were missed during my pregnancy care	8	*36*	10	*29*
I got sufficient information during pregnancy about breastfeeding	12	*55*	27	*79*
I got adequate information during my pregnancy about labour and birth	17	*77*	31	*91*
I got sufficient information during my pregnancy about caring for myself after the birth	11	*50*	21	*62*
I got adequate information during my pregnancy about community services	12	*55*	23	*68*
I was informed about how long I would stay in hospital after the birth	15	*68*	27	*79*

^a^The statements provided here had options from ‘Strongly disagree’, ‘Disagree’, ‘Unsure’, ‘Agree’ to ‘Strongly agree’, and here the ‘Agree’ and ‘Strongly agree’ responses are added and presented

### Overall care in pregnancy

Three quarters of women (17/22) in usual care rated their overall care in pregnancy as ‘6’ or ‘7’ on a 7-point scale where ‘7’ was ‘Very good’, as did 88% of women (30/34) in Group Care. A higher percentage of women in usual care felt prepared for labour and birth compared with those in Group Care (62%, 13/21, vs 44%, 15/34).

### Maternal and infant outcomes at post birth telephone interview

In the first week at home with their new baby, 41% (9/22) of women in usual care and 38% (13/34) of women in Group Care rated their confidence looking after the new baby as a ‘6’ or ‘7’ on a scale of ‘1’ to ‘7’, where ‘1’ was ‘Not at all confident’ and ‘7’ was ‘Very confident’. Regarding preparation for baby care and parenting, 59% (13/22) of women in usual care felt prepared for baby care and parenting (‘6’ or ‘7’), compared with 29% (10/34) in Group Care.

When asked if their current health was ‘Poor’, ‘Fair’, ‘Good’, ‘Very good’, or ‘Excellent’, 77% (17/22) of women in usual care and 71% (24/34) of women in Group Care rated their health as ‘Very Good’ or ‘Excellent’. One of the ten items in the EPDS was accidentally omitted (*I have been so unhappy that I have had difficulty sleeping*), so we used the validated 5-question version – EPDS-Dep-5 [23] to report on here. The EPDS-Dep-5 score is calculated using five questions from the original EPDS—‘*I have been able to laugh and see the funny side of things*’, ‘*I have looked forward with enjoyment to things*’, ‘*I have felt sad or miserable*’, ‘*I have been so unhappy that I have been crying*’, and ‘*The thought of harming myself has occurred to me*’ (Cronbach’s alpha 0.82 compared to 0.88 for the full EPDS). A score of four or greater is considered indicative of potential clinical depression when using the EPDS-Dep-5. Here, 9% (2/22) of women in usual care scored above 4, compared with 15% (5/34) in Group Care.

### Infant feeding

At the post birth interview (8.7 weeks postpartum on average), 85% (29/34) in Group Care and 95% (21/22) in usual care were giving *any* breast milk in the previous 24 h, 56% (19/34) in Group Care and 73% (16/22) in usual care were giving *only* breast milk in the previous 24 h, and 32% (11/34) in Group Care and 45% (10/22) in usual care had given only breast milk since birth. When asked how long it was before women felt confident with breastfeeding, on average, women felt confident after 12 and 15 days respectively for usual care and Group Care. Five women in Group Care and two in usual care stated that they still did not feel confident with breastfeeding or never felt confident with breastfeeding.

At 1 week post birth, 55% of those in usual care and 62% in Group Care felt confident caring for their baby. By 1 month, the majority from both groups (usual care 92%, Group Care 93%) felt confident caring for their baby.

### Women’s views of Group Care (intervention group only)

Overall, women were extremely positive about the Group Care model as shown in Table 6. Very few (6%, 2/33) preferred a women-only group. Most women were happy with when their group sessions were held, with only 21% (7/33) stating that they would have preferred a different time or day, and 36% (12/33) would have preferred to have group sessions on a weekend. Just over half (53% 17/32) would be happy to attend Group Care in a community centre near where they lived.

**Table 6:** Women’s views of Group Ccare

Statement	Agree/strongly agree^a^	
	*n*	*%*
	(*n* = 33)	
I enjoyed having GEM care for my pregnancy	32	*97*
I was comfortable with other people’s partners being at the GEM sessions	32	*97*
I would recommend my friends to have GEM care at the Women’s	31	*94*
Meeting other mothers is important to me	30	*91*
I felt free to discuss sensitive issues privately with the midwives	30	*91*
I felt free to discuss sensitive issues within the group	24	*73*
I would have GEM care for my next pregnancy (*n* = 32)	22	*69*
I have made friends with other people from my GEM group	19	*58*
I would have preferred my GEM Care group to include women who have had babies previously	9	*27*
I would have preferred to attend GEM sessions that were women only	2	*6*

^a^The statements provided here had options from ‘Strongly disagree’, ‘Disagree’, ‘Unsure’, ‘Agree’ to ‘Strongly agree’, and here the ‘Agree’ and ‘Strongly agree’ responses are added and presented

Just over half the women (55% 18/33) felt that they had enough alone time with the midwife during the group sessions. Over half (55% 18/33) missed at least one session, with the main reasons being ill-health or birth of the baby prior to the final session. Most women (91% 30/33) attended the additional weekend session that focused just on education. All found it useful, and almost all (87%; 26/30) attended with their partner or support person.

### Midwives’ views

Seven of the eight group care midwives attended a focus group conducted by RM and RH in November, 2017. They were aged from 25 to 44 years and had between 2 and 7 years midwifery experience post qualification. The midwives were highly motivated to try a new model of care and to try and improve care for women. They spoke of their frustrations with routine care and how they felt Group Care could be a better option for themselves and women. They wanted to work in a way that would ‘build connections’ and ‘empower women’, and felt that Group Care enabled this. These became one of the overarching themes, labelled *Connections and empowerment*. This emerged from two sub-themes—‘*a different quality of relationship*’, and ‘*a more meaningful midwifery experience*’, which were both related to how the midwives’ experienced the model, and how it made them feel. The other overarching theme, ‘*Operational aspects*’, was more related to the functioning of the model. The themes are described in more detail below [24].

### Connections and empowerment

#### A different quality of relationship

Only one of the midwives had worked in a continuity model before, so for most, this was their first experience of continuity of care, and they found it extremely positive and very woman-centred.

The continuity was not just with the women, but with each other and the students they worked with. The midwives spoke highly of the students’ involvement and how much satisfaction they got from working with them and being able to provide a learning environment that both enabled continuity and enhanced skill development.

*You know what [students] can do and what they can’t do and so they actually get a lot more from the experience* (MW4, age 26, 2 years’ experience).

The midwives found themselves becoming invested in the women’s well-being. They spoke of the women’s experience and how much that meant to them.

*… once, you know, really make a connection with a woman… you’re like, oh my God, she’s so upset. What can I do for her?* (MW7, age 30, 6 years’ experience).

The midwives were positive about building connections with the women and the women’s connections with each other. They thought that the relationships between the women would empower them in their decision-making and increase the ownership in their care.

*I just believe in the power of women getting to know each other and sharing their stories… where that can lead is pretty extraordinary* (MW2, age 44, 4 years’ experience).

The midwives developed strong relationships with each other that enhanced trust, and meant that they could learn from each other. That empowered themselves as midwives.

*One of the biggest benefits for me was being able to work so closely with another midwife and that was a real highlight … we work in teams every day but we don’t work side by side [in standard care]. I learnt a lot from [the other midwife] and I feel that she learnt a bit from me… just building that rapport… and knowing that we’re both the same, it really felt amazing. It just really worked… There’s two of you and you’re both committed and…you can be completely different and come from different angles…but … you can built that trust with each other* (MW1, age 34, 7 years’ experience).

#### A more meaningful midwifery experience

The midwives spoke of how fulfilled they felt by the model, the experience that they got out of it, and how it had affected their practice and given their work more meaning.

*I felt like it ran really well. I felt like I was on … a very steep learning curve in facilitating – and I feel like I sort of got it a bit and I just feel like I’d like to just do another group …It’s a good investment. Like it was honestly the highlight of my job for four months…It’s not like you’re leaving and you’re like oh my God, that was like a really draining day or anything. You’re like, oh like it so amazing* (MW6, age 27, 3 years’ experience).

*Operational aspects* were also very salient. This was made up of four themes: *Communication*, *The model* (related to functional issues), *Midwife preparation and training*, and *Administration*.

#### Communication

The midwives found that there was a lack of knowledge of this new model of care throughout the hospital and that medical staff would book extra unnecessary appointments. Decisions were made about women’s care without consulting the group midwives because the medical team did not understand or were not aware of the model.

*I found with doctors was that they weren’t aware of what GEM was…you’d have to constantly just explain the concept* (MW4, age 26, 2 years’ experience).

#### The model

The midwives liked the length of the sessions and how much time they had to get to know the women—time to make a connection and time to facilitate education.

*…one of the biggest issues that I have with the standard model is that you do have about two minutes to go through very important information* (MW4, age 26, 2 years’ experience).

*Two hours is a massive amount of time for every session with them so I think they got a lot out of it* (MW7, age 30, 6 years’ experience).

The groups were run in two different spaces. One was a large clinic area with separate areas for the group and for pregnancy checks. The other space was a large group room with a bed behind a privacy screen, located near the door. The latter room was perceived to have issues with the space and maintaining privacy. Midwives voiced concerns that it may have affected their connections with women.

[while doing the checks] *… really hard to have any ‘heart to heart’ discussion. The only time was … if you could talk [the woman] a bit quietly while the group was making a lot of noise* (MW6, age 27, 3 years’ experience).

*I felt like there was some things that the women didn’t tell us [because of the lack of privacy in the room]* (MW1, age 34, 7 years’ experience)

Partners or support people were invited to be part of all the sessions, but for daytime sessions less partners attended. The midwives considered that the positive aspects of partners/support people being present were inclusiveness, connections, and support.

*…they formed friendships with the other partners and with the women as well…* (MW5, age 27, 3 years’ experience).

*… our dads were really supportive and it really blew me away…you get the dads and mums to write what they wanted to know and…dad’s like number one is [about] breastfeeding* (MW1, age 34, 7 years’ experience).

Negative aspects of having partners in the sessions were some concerns about women being about to disclose sensitive issues. Some felt that some of the session should have been women only.

*…a session … that was women only [would have been good]* (MW2, age 44, 4 years’ experience).

Some found that groups with all partners did not bond as much as other groups that had only a few partners.

#### Midwife preparation and training

The midwives that did the workshop felt really prepared; however, two that missed out felt under-prepared and experienced difficulties with how it would flow and work:

*… [I did the reading and] I got gist of what it was about but I think was so much more [in the face-to-face training]* (MW6, age 27, 3 years’ experience).

#### Administration

One of the biggest struggles was finding time for the administrative tasks. Documentation, following up results, booking medical appointments all took several hours, and were not included in the allocated session time. Midwives followed up communication from the women in their own time. While the workload of this was a negative, they wanted to make sure issues were resolved and felt responsible for the women’s care.

*Like you feel connected to them as well and you're really interested to see how they go. You really want to follow them up* (MW3, age 25, 4 years’ experience).

Overall, the midwives were very positive about the experience and very keen to repeat it. All said they would work in the model again and thought that the model should be expanded. Two felt so strongly about it that they requested to do the training for other midwives to make the model more sustainable.

## Discussion

Our pilot RCT of Group Care addressed all our study aims and demonstrated feasibility for a larger RCT, and acceptability for women and midwives. Thirty-five percent of women approached agreed to participate, very similar to other successful studies from our team [25] and to a recent pilot RCT of Group Care in the UK [26]. No woman declined participation because she would be randomised, and none declined because other women’s partners would be present in the group setting. Any women who declined did so more for reasons aligned with simply preferring a different model of care.

In terms of the clinical outcomes, there was no hypothesis testing for between group differences. We explored our ability to obtain clinical outcome data for the larger RCT and this was achieved. We found no sign of obvious harm in women allocated to Group Care. Of women allocated to usual care, 22% had a caseload (known) midwife model (higher than usual at the site—normally 7% have access to caseload), 25% had team midwifery (eight midwives working in a team to care for a group of women), 50% had standard midwifery care (no continuity), and one woman had medical care only. Rates of CS were similar by group—a likely explanation is the high percentage of women in usual care receiving midwifery continuity with the associated better outcomes (far higher than will be possible in women allocated to usual care in the larger group care RCT). There was no issue accessing our clinical outcome data. A higher percentage of women allocated to Group Care reported that their partner/support person attended with them, and likewise, a higher percentage was positive about the various aspects of information they received.

The women allocated to the Group Care intervention were very satisfied with the care they received in the group setting, and happy with the information provision, consistent with the Cochrane Review [3], and the pilot study of the UK RCT [14, 26], where the model was acceptable to women and enhanced their experiences [27]. Just under half attended all six 2-h sessions, slightly higher than the recent UK pilot, where 37% attended six or more sessions (although that was in a context of eight group sessions) [26]. Of the remainder, most (94%) missed only one or two sessions, with the main reasons being ill-health or birth of the baby prior to the final session, and we do not expect that this is very different from women attending maternity care generally. Most women also attended the additional weekend session that focused just on education. The vast majority (94%) enjoyed the Group Care model, 91% would recommend it to their friends, and 88% said that meeting other mothers was important to them. At least two of the groups continued to meet for a year or two after their infant’s birth, and invited their respective midwife facilitators, further evidence of positive outcomes of the model. Almost all women brought a support person with them to the groups, and very few preferred women-only sessions. Most (82%) felt comfortable having care with other people’s partners present, and 55% had enough alone time with their midwife. Most (71%) felt free to discuss sensitive issues within the group, and 88% to have private discussions with the midwife. Just over a third would have liked the option of their sessions being held on the weekend, and just over half would be happy to attend a community-based setting—and these are aspects we can plan for in the larger study. These were some of the key issues we wanted to understand when we planned the pilot, so consider that this information provides evidence of the viability of the larger RCT, as well as guidance to the areas we would need to adjust in the subsequent study.

Eight midwives provided Group Care. All were very positive, consistent with other studies [8, 9], and wanted to continue working in the model. They found the work fulfilling, valued the continuity, and felt more invested in women’s experiences. One of the issues was finding time for the administrative tasks, and this has been found elsewhere, where midwives reported that having protected time, training, and ongoing support was essential [9], and that there needs to be further exploration on midwives’ workload, tasks, and structural supports [28]. The group setting and session length allowed women to share their experiences and build connections with the midwives and each other. We had no problem recruiting midwives to the model, and no issues with allocating times for sessions— again, reassurance about the potential viability of the larger RCT.

Our pilot intervention was tested in a pre-COVID-19 context, and thus, groups were conducted face-to-face, which is considered the best option [9]. There is evidence, however, that if necessary, we could conduct a larger RCT with some groups conducted online. Recruitment to the current UK RCT [14] was paused in 2020, with women already recruited and allocated to group care returned to having one-to-one care, but where possible group elements for some participants were retained, and a number of the sites reported successfully implementing the group care model online [9, 14]. This followed the group’s successful pilot RCT which was very similar to our own [26]. The research team recommenced recruitment in May 2022 with maximum flexibility between in person care and remote elements as required, and continuing to recruit in July 2023 (Christine McCourt, personal communication, May 2022, July 2023). The positives of this approach were noted—such as the opportunity to break down barriers such as geography and childcare, while at the same time still providing the peer support [9], findings similar to those reported in the Netherlands [29].

### Strengths and weaknesses

This was a carefully conducted pilot RCT that allowed us to test all our processes and explore feasibility. Our pilot study was not powered to explore differences in clinical outcomes; thus, we have made no comment on any minor differences in percentages by group, regardless of the direction of the outcome by group. Likewise, while there were some percentage differences in women’s ratings of care (such as women in Group Care rating doctor’s appointments as more rushed), we cannot make any conclusions regarding this, and would explore such issues more fully in a larger study.

## Conclusion

Group Care targets key outcomes that have been difficult to tackle globally, and which remain major public health issues, including the high (and increasing) CS rate, the rate of preterm and low birthweight infants, and the increasing proportion of women who suffer anxiety related to pregnancy and birth. All these can have negative short- and long-term implications for women, infants, and families.

Given our various findings, we conclude that our pilot RCT has demonstrated the feasibility of conducting a large adequately powered RCT of group care in the Australian context. We developed the Group Care schedule and showed feasibility of all processes. We found that women were interested in participating and willing to be randomised, and that there was a high proportion of women in Group Care who attended the majority of sessions. We confirmed our ability to extract key clinical outcome data for analysis and confirmed the findings of the Cochrane review of group antenatal care—that there was no obvious adverse outcomes [3], and the model was perceived very positively by women [3] and midwives [8]. Women were willing to complete the postpartum questionnaire. We found minor issues that we would adjust for the larger RCT, mostly related to timing and location of sessions.

We therefore plan to proceed, and design a large adequately powered RCT to test the safety and efficacy of Group Care (compared to one-to-one care). In light the COVID-19 pandemic context, we will follow the UK example, and design an RCT with the intervention as per our pilot, but ensure that the model can be flexible if the need arises, while maintaining as many elements of group care as possible [12], and provide all women allocated to the intervention solely group-based care whenever possible.

### Supplementary Information

Supplementary material 1.

## Data Availability

The data analysed and presented in this paper are available from the first author upon reasonable request.

## Abbreviations

CS: Caesarean section
EPDS: Edinburgh Postnatal Depression Scale
Group care: Group-based antenatal care and education
RCT: Randomised controlled trial

## References

[1] Sandall J, Tribe R, Avery L, Mola G, Visser G, Homer C (2018). Optimising caesarean use 2. Lancet.

[2] Blencowe H, Lee A, Cousens S, Bahalim A, Narwal R, Zhong N (2013). Preterm birth–associated neurodevelopmental impairment estimates at regional and global levels for 2010. Pediatr Res.

[3] Catling CJ, Medley N, Foureur M, Ryan C, Leap N, Teate A (2015). Group versus conventional antenatal care for women. Cochrane Database Syst Rev..

[4] Sandall J, Soltani H, Gates S, Shennan A, Devane D (2016). Midwife-led continuity models versus other models of care for childbearing women. Cochrane Database Syst Rev..

[5] Chen I, Opiyo N, Tavender E, Mortazhejri S, Rader T, Petkovic J (2018). Non-clinical interventions for reducing unnecessary caesarean section. Cochrane Database Syst Rev..

[6] Cohen S, Underwood L, Gottlieb BE (2002). Social support measurement and intervention: a guide for health and social scientist.

[7] Lathrop B (2013). A systematic review comparing group prenatal care to traditional prenatal care. Nurs Women’s Health.

[8] Teate A, Leap N, Homer C (2013). Midwives’ experiences of becoming CenteringPregnancy facilitators: a pilot study in Sydney. Women Birth.

[9] Wiseman O, Emmett L, Hickford G, Knight M, Lazar J, Yuill C (2022). The challenges and opportunities for implementing group antenatal care (‘Pregnancy Circles’) as part of standard NHS maternity care: a co-designed qualitative study. Midwifery..

[10] Carter E, Temming L, Akin J, Fowler S, Macones G, Colditz G (2016). Group prenatal care compared with traditional prenatal care. a systematic review and meta-analysis. Obstetrics Gynecol..

[11] Byerley BM, Haas DM (2017). A systematic overview of the literature regarding group prenatal care for high-risk pregnant women. BMC Pregnancy Childbirth.

[12] Sharma J, O’Connor M, Rima JR (2018). Group antenatal care models in low- and middle-income countries: a systematic evidence synthesis. Reprod Health.

[13] Carter EB, Barbier K, Sarabia R, Macones GA, Cahill AG, Tuuli MG (2017). Group versus traditional prenatal care in low-risk women delivering at term: a retrospective cohort study. J Perinatol.

[14] Wiggins M, Sawtell M, Wiseman O, McCourt C, Eldridge S, Hunter R (2020). Group antenatal care (Pregnancy Circles) for diverse and disadvantaged women: study protocol for a randomised controlled trial with integral process and economic evaluations. BMC Health Serv Res.

[15] van Zwicht B, Crone M, van Lith J, Rijinders M (2016). Group based prenatal care in a low- and high-rsik population in the Netherland: a study protocol for a stepped wedge cluster randomized controlled trial. BMC Preg Childbirth.

[16] Chen L, Crockett AH, Covington-Kolb S, Heberlein E, Zhang L, Sun X (2017). Centering and Racial Disparities (CRADLE study): rationale and design of a randomized controlled trial of centeringpregnancy and birth outcomes. BMC Pregnancy Childbirth.

[17] World Health Organization. WHO recommendations on antenatal care for a positive pregnancy experience Geneva2016 [Available from: https://apps.who.int/iris/bitstream/handle/10665/250800/WHO-RHR-16.12-eng.pdf;sequence=1. 28079998

[18] Forster DA, McLachlan HL, Davey MA, Biro MA, Farrell T, Gold L (2016). Continuity of care by a primary midwife (caseload midwifery) increases women’s satisfaction with antenatal, intrapartum and postpartum care: results from the COSMOS randomised controlled trial. BMC Pregnancy Childbirth.

[19] McLachlan H, Forster D, Davey M-A, Farrell T, Flood M, Shafiei T (2016). The effect of primary midwife-led care on women’s experience of childbirth: results from the COSMOS randomised controlled trial. BJOG. Accepted September 2015. BJOG..

[20] Cox J, Holden M, Sagovsky R (1987). Detection of postnatal depression: development of 10-item Edinburgh Postnatal Depression Scale. Br J Psychiatry.

[21] Harris PA, Taylor R, Minor BL, Elliott V, Fernandez M, O'Neal L, McLeod L, Delacqua G, Delacqua F, Kirby J, Duda SN. REDCap Consortium. The REDCap consortium: Building an international community of software platform partners. J Biomed Inform. 2019;95:103208. 10.1016/j.jbi.2019.103208. Epub 2019 May 9. 10.1016/j.jbi.2019.103208 PMC7254481 31078660

[22] StataCorp (2015). Stata Statistical Software: release 14. College Station.

[23] Harel D, Levis B, Ishihara M, Levis A, Vigod S, Howard L (2021). DEPRESsion Screening Data (DEPRESSD) EPDS Collaboration. Shortening the Edinburgh postnatal depression scale using optimal test assembly methods: development of the EPDS-Dep-5. Acta Psychiatr Scand.

[24] Forster D, Matthews R, Hyde F, Llwellyn F, Shafiei T, Newton M, et al. The FUSCHIA study. FUture proofing the midwifery workforce in Victoria: a statewide cross-sectional survey exploring Health, wellbeing and SustaInAbility. La Trobe University. 2021.

[25] Forster DA, Moorhead AM, Jacobs SE, Davis PG, Walker SP, McEgan KM (2017). Advising women with diabetes in pregnancy to express breastmilk in late pregnancy (Diabetes and Antenatal Milk Expressing [DAME]): a multicentre, unblinded, randomised controlled trial. Lancet.

[26] Sawtell M, Wiggins M, Wiseman O, Mehay A, McCourt C, Sweeney L (2023). Group antenatal care: findings from a pilot randomised controlled trial of REACH Pregnancy Circles. Pilot Feasibility Stud.

[27] Hunter LJ, Da Motta G, McCourt C, Wiseman O, Rayment JL, Haora P (2019). Better together: a qualitative exploration of women’s perceptions and experiences of group antenatal care. Women Birth.

[28] Lazar J, Boned-Rico L, Olander EK, McCourt C (2021). A systematic review of providers’ experiences of facilitating group antenatal care. Reprod Health.

[29] Rijnders M, Jans S, Groesen K (2021). Centering in times of the COVID-19 pandemic. Pract Midwife.

