# Supplementary material for: Exploring the feasibility of conducting a randomised controlled trial of group-based pregnancy care and education: a pilot randomised controlled trial in Melbourne, Australia
Source: Pilot Feasibility Stud. 2024 May 20;10:81. doi: 10.1186/s40814-024-01501-8 (PMC11103971; doi:10.1186/s40814-024-01501-8)
Supplement: Supplementary file 1 — Supplementary material 1. [file 40814_2024_1501_MOESM1_ESM.docx]

**Supplementary table: Schedules for information content and session focus by visit^#^**

| **Gestation** | **Usual care: Women’s contact hours in pregnancy** | **Gestation** | **Group antenatal care and education: Women’s contact hours in pregnancy** |
| --- | --- | --- | --- |
| All women attended Booking in MW visit 40 minutes (18 weeks) and Dr visit 15 minutes (20 weeks) | | | |
| 26 weeks | MW clinical visit 20 minutes | 26 weeks | 2 hours MW clinical and education |
| 30 weeks | MW clinical visit 20 minutes | 30 weeks | 2 hours MW clinical and education |
| 33 weeks | MW clinical visit 20 minutes | 32 weeks | 2 hours MW clinical and education |
| Approx.  30-34 weeks | 7 hours education and hospital tour including partners/support people* | 34 weeks | 5.5 hours MW education and hospital tour including partners/support people (no clinical component) |
| 36 weeks | Dr visit 10 minutes | 36 weeks | Dr visit 10 minutes |
| 38 weeks | MW clinical visit 20 minutes | 38 weeks | 2 hours MW clinical and education |
| 40 weeks | MW clinical visit 20 minutes | 40 weeks | 2 hours MW clinical and education |
| 41 weeks | Dr visit 10 minutes | 41 weeks | Dr visit 10 minutes |
| **Total:** | Dr 35 minutes  plus Midwives 9 hours 20 minutes = **9 hrs 55 minutes contact**  No continuity of carer except if in a model that provides continuity | **Total:** | Dr 35 minutes plus Midwives 16 hours 10 minutes =  **16 hours 45 minutes contact**  Includes continuity of carer for 15 hours 30 mins |

* *Usual care* – *separate childbirth education (usually with an associated fee)*
